# Ebola Epidemic — Liberia, March–October 2014

**Published:** 2014-11-21

**Authors:** Tolbert Nyenswah, Miatta Fahnbulleh, Moses Massaquoi, Thomas Nagbe, Luke Bawo, James Dorbor Falla, Henry Kohar, Alex Gasasira, Pierre Nabeth, Sheldon Yett, Bernadette Gergonne, Sean Casey, Benjamin Espinosa, Andrea McCoy, Heinz Feldman, Lisa Hensley, Mark Baily, Barry Fields, Terrence Lo, Kim Lindblade, Josh Mott, Lucy Boulanger, Athalia Christie, Susan Wang, Joel Montgomery, Frank Mahoney

**Affiliations:** 1 Ministry of Health and Social Welfare, Liberia; 2 World Health Organization; 3 United Nations Children’s Fund; 4 Médecins Sans Frontières; 5 International Medical Corps; 6 US Navy; 7 National Institutes of Health; 8 US Army Medical Research Institute of Infectious Diseases; 9 CDC

On March 21, 2014, the Guinea Ministry of Health reported the outbreak of an illness characterized by fever, severe diarrhea, vomiting and a high fatality rate (59%) (1), leading to the first known epidemic of Ebola virus disease (Ebola) in West Africa and the largest and longest Ebola epidemic in history. As of November 2, Liberia had reported the largest number of cases (6,525) and deaths (2,697) among the three affected countries of West Africa with ongoing transmission (Guinea, Liberia, and Sierra Leone) (2). The response strategy in Liberia has included management of the epidemic through an incident management system (IMS) in which the activities of all partners are coordinated (3). Within the IMS, key strategies for epidemic control include surveillance, case investigation, laboratory confirmation, contact tracing, safe transportation of persons with suspected Ebola, isolation, infection control within the health care system, community engagement, and safe burial. This report provides a brief overview of the progression of the epidemic in Liberia and summarizes the interventions implemented.

The data sources used to describe the epidemic included aggregate situation report data reported daily from counties to the Liberian Ministry of Health and Social Welfare (MOHSW), data from Ebola treatment units (ETUs), and Ebola laboratory test data. Case definitions used by the Liberian MOHSW have been described previously (4). Field investigative reports from rapid response teams deployed in 12 counties during October 25–November 5 also were reviewed.

ETU admission records included all patients admitted to ETUs in Liberia. Non-Ebola patients were defined as those admitted to ETUs but released based on documentation of two consecutive negative Ebola reverse transcription–polymerase chain reaction (RT-PCR) tests at least 72 hours apart. Ebola cases were defined as illnesses in patients who did not have documentation of a negative RT-PCR test result even if laboratory results were not recorded (i.e., including confirmed [those with ETU documentation of a positive RT-PCR result], and those probable and suspected Ebola patients for whom there were no ETU documentation of negative RT-PCR results). Seven percent of Ebola cases did not have laboratory results.

Confirmation of Ebola in the laboratory was undertaken by real-time PCR by one laboratory in Lofa County, three laboratories in Montserrado County, and one laboratory in Bong County. Results were available for 7,043 specimens representing 5,132 patients. Because patient-level identifiers were not assigned consistently, a unique identifier was created using patient initials, sex, age, and home location to link multiple specimen records for the same patient. A total of 413 specimens (<6%) lacked the information necessary for unique identifier assignment and were excluded. Test week was based on the first specimen taken. Information on the number of safe burial teams trained and operational, by county, was obtained from Global Communities and the International Federation of Red Cross and Red Crescent Societies, nongovernmental organizations (NGOs) contracted to collect human remains.

In March 2014, Ebola virus infection was detected in Lofa County in a patient returning from an outbreak area in Guinea. During March–October, 2014, Liberia counties reported 2,445 suspected, 1,623 probable, and 2,456 confirmed Ebola patients to MOHSW. In the months following detection in Lofa, the county experienced multiple waves of outbreaks, with a peak in case counts between late July and late September (Figure 1). In June 2014, the first cases were detected in densely populated Montserrado County (estimated pop. 1.5 million), leading to a countywide outbreak that peaked in late September, followed by a rapid decrease in the Ebola case counts since that time. Concurrent with the Montserrado County outbreak, cases were identified from all counties in Liberia, with 12 of 15 counties reporting cases to MOHSW during October 25–November 3 (Figure 2).

As the Lofa outbreak expanded, many suspected Ebola patients sought assistance within the national health care system, leading to multiple outbreaks among health care workers throughout the country (5). Many health care facilities closed, and health care workers refused to come to work. Those facilities that remained open provided limited care. As of November 8, MOHSW reported 329 health care workers infected with Ebola.

Providing care to this large number of patients has been a challenge. In April, MOHSW with support from Médecins Sans Frontières (MSF) established an isolation facility at an old refugee transit facility in the town of Foya, where the disease was first detected. The Firestone company established a 23-bed unit in April in Margibi county (4). In Montserrado, an NGO converted a hospital chapel (ELWA1) into an isolation facility. After health care workers on staff had acquired infections, the NGO withdrew from Liberia, and management of the facility was transferred to MSF and the Liberian government. As the outbreak intensified in Montserrado in July, patients from ELWA1 were moved to an outpatient department (ELWA2) within the hospital grounds. With an overwhelming demand for beds, the government converted a cholera ward at JFK Hospital into a treatment unit while MSF started construction on a new site, ELWA3, which opened August 17. The initial admissions to this unit included a large number of critically ill patients who were waiting outside the ETUs seeking care.

In response to the continued demand, the government, the World Health Organization, and the World Food Programme began construction on two additional ETUs in Monrovia, and the partners began planning construction on multiple ETUs throughout the country. In collaboration with Save the Children, the International Medical Corps opened the Bong County ETU on September 15. Island Clinic ETU was opened as a 120 bed unit on September 21 and was immediately filled with approximately 200 patients. The Liberian Ministry of Defense ETU opened as a 100-bed unit on November 6 (Figure 1). By November 8, there were a total of 697 beds available in nine ETUs in Liberia. During June 5–November 8, 2014, a total of 4,025 patients were admitted to ETUs in Liberia; 2956 were classified as having Ebola (73.5%).

To prevent transmission associated with funeral practices, safe burial teams were trained in all counties, beginning in Lofa with the opening of the Foya ETU, followed by training of burial teams in Montserrado. Despite reported community resistance, the government implemented a program to cremate all remains in Montserrado County in response to the large number of human remains. In September, there was a significant expansion of safe burial teams in counties, with 54 teams trained and equipped nationwide by October 5, up from fewer than 10 in August.

From October 25 to November 3, MOHSW field teams initiated seven rapid response investigations outside of Montserrado County in hard-to-reach areas (Figure 2). These responses involved the critical support of international partners including the World Health Organization, United Nations Children’s Fund, Emergency-Health, Peace Corp staff, and CDC. These investigations represented increased recognition of ongoing outbreaks in hard-to-reach areas, and also reflected ongoing transmission of Ebola in Grand Cape Mount, Grand Kru, Grand Bassa, Gbarpolu, Bomi, and Sinoe counties within a recent 1-week window. Many of the communities are remote, with three not accessible by road. Activities to interrupt transmission in these areas include the rapid establishment of triage and isolation strategies to treat patients in place (in remote areas) or to transport patients to the nearest ETU. Response teams also worked to enhance contact tracing and active surveillance, social mobilization, and infection control practices.

In June and July, <50 specimens were tested per week, with the proportion of specimens testing positive ranging from 30% to 70%. With increases in laboratory capacity, the number of specimens tested increased to approximately 500 per week through August and early September. The peak number of specimens tested occurred in late September and early October at approximately 700 per week with approximately 70% of specimens testing positive at that time. Despite increases in laboratory capacity, there has been a decrease in the number of tests performed since September 28. Overall, out of 5,132 patients who could be identified from the laboratory data, 2,941 (57.3%) tested positive (Figure 3).

What is already known on this topic?

The current Ebola epidemic in West Africa is the largest ever reported, and Liberia has experienced the largest number of cases. Previous outbreaks of Ebola have been controlled through early identification of cases through contact management and health care system preparedness, isolation and treatment of patients, social mobilization, and safe burials.

What is added by this report?

Data from Ebola treatment units, laboratories, and daily situation reports were analyzed to describe the course of the epidemic in Liberia and the recent geographic distribution of cases. There has been a decrease in cases since mid-September, and the initiation of interventions might have played an important role in the decline. However, Ebola continues to spread in at least 12 of 15 Liberian counties and focal outbreaks in hard-to-reach areas are now frequent.

What are the implications for public health practice?

Although Ebola cases are declining in Liberia, the increased geographic distribution of cases along with outbreaks in remote areas are likely to require an increase in the level of intervention before Ebola can be eliminated.

## Discussion

The outbreak of Ebola in Liberia is complex and evolving. Trends in this analysis are based on ETU admissions and laboratory data and might underestimate case counts among persons who do not get tested, among persons who do not seek care in ETUs, and among persons in areas without ETUs. Given these limitations, the available data indicate Liberia and its partners have made significant strides in possibly reducing Ebola transmission in at least Lofa and Montserrado counties. In particular, there has been a significant decline in case counts with increased bed capacity, safe patient transport, training of burial teams, and ongoing social mobilization and community-led interventions. However, progress of control efforts is tenuous and will require rapid response to multiple outbreaks, improved infection control throughout the health care system, and extensive community engagement to stop transmission. The widespread distribution of disease in urban and rural settings coupled with a highly mobile population, presents extraordinary challenges. Intensified case identification and contact tracing efforts are needed in all counties while sustaining current interventions and refining control strategies to stop transmission in other counties.

Controlling the epidemic in counties outside of Monrovia will require construction of ETUs in all counties, along with increases in capacity for specimen transport and testing networks. Early case recognition, identification, and isolation, along with contact identification and management, are needed to rapidly contain focal outbreaks in hard-to-reach and newly affected areas. Addressing these focal outbreaks will demand intensified and flexible support, including special attention to social mobilization and community engagement. These increases in response activities must counter any potential relaxation of control measures within the IMS response and will require an effective surveillance system and continued support from the international community. Adapting control strategies to the epidemic and rapidly expanding response activities are essential to prevent endemic Ebola transmission and international spread.

## Figures and Tables

**FIGURE 1:**
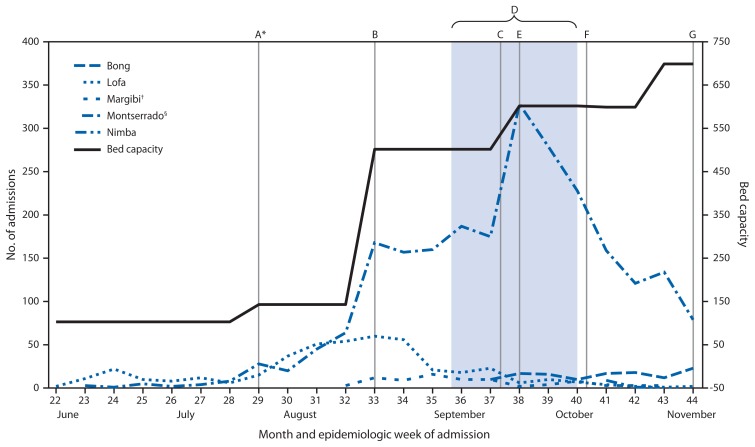
Number of patients admitted to Ebola treatment units (ETUs), by county and week — Liberia, June 5–November 1, 2014 **Abbreviation:** ETU = Ebola treatment unit. * A) opening of ELWA2 ETU, B) opening of ELWA3 ETU and JFK ETU, C) opening of Bong ETU, D) safe burial teams trained and deployed in all counties, E) opening of Island Clinic ETU, F) opening of Nimba ETU, G) opening of MoD ETU. Not shown: openings of ELWA 1 ETU (April 2014), Margibi ETU (April 2014), and Lofa ETU (April 2014). ^†^ Margibi ETU opened in April but had no cases until August 8. No data were reported for October 1–21, 2014. ^§^ Includes JFK, Island, ELWA-2, ELWA-3, and MoD ETUs.

**FIGURE 2:**
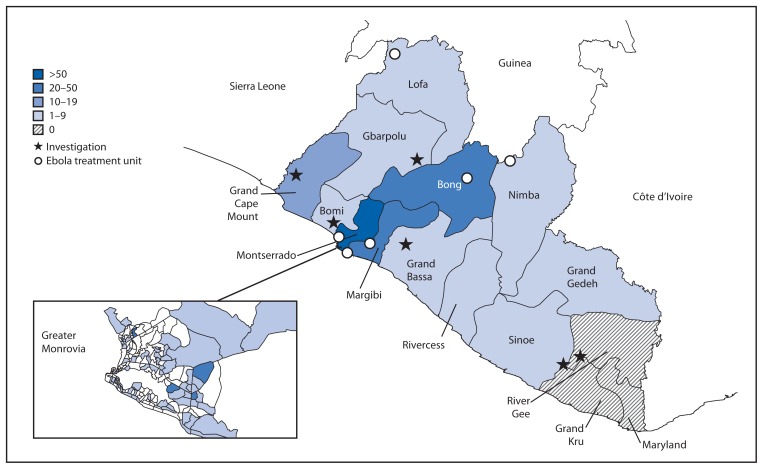
Number of suspected and probable Ebola cases from MOHSW daily situation reports and locations of rapid response investigations,* by county, with inset showing case distribution in greater Monrovia — Liberia. October 25–November 3, 2014 **Abbreviation:** MOHSW = Liberian Ministry of Health and Social Welfare. * Grand Cape Mount County (Jene Wonde): 24 unexplained deaths; most recent probable or confirmed case on November 5; Grand Kru County (Parluken and Niaplapko): 21 deaths and 14 probable or confirmed cases; most recent probable or confirmed case on November 3; Grand Bassa County (John Logan Town): 17 unexplained deaths including two confirmed Ebola cases and one probable case; most recent confirmed case on October 25; Gbarpolu County (Geleyansiesu) : Six probable and 12 confirmed cases, including eight deaths, most recent confirmed case on November 6; Bomi County (Dorley-La and Gbah): Three confirmed and four probable cases, including five deaths; most recent confirmed case on November 3; Sinoe County (Government Camp): Three confirmed cases since October 31.

**FIGURE 3:**
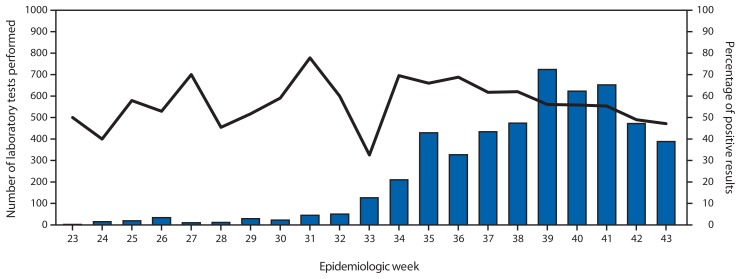
Number of laboratory tests performed and percent positive for Ebola, by week — Liberia, June 5–November 1, 2014.
